# Relation Between Physicians’ Emotional Response and Stigma Around Suicide

**DOI:** 10.1192/j.eurpsy.2025.514

**Published:** 2025-08-26

**Authors:** R. T. Monteiro, P. B. Bolzan, T. P. Comissoli, G. I. Zanella, Y. A. Ferrão

**Affiliations:** 1 Universidade Federal de Ciências da Saúde de Porto Alegre; 2 Serviço de Psiquiatria, Hospital Materno Infantil Presidente Vargas (HMIPV), Porto Alegre, Brazil; 3 Vrije Universiteit Amsterdam, Amsterdam, Netherlands

## Abstract

**Introduction:**

Suicide is a global health issue. Clinicians still have difficulties to differentiate patients who will or not commit suicide. This process is influenced by emotional and rational factors. Emotional responses (also known as countertransference) refers to what emotions clinicians experience. When working with suicidal patients, clinicians frequently experience negative emotions, such as fear, guilt and hopelessness. Clinicians’ negative emotions responses contribute to their assessment of risk. Possible factors that influence emotional responses are myths and beliefs around suicide, contributing to stigma.

**Objectives:**

This study aims to investigate the relationship between emotional responses, knowledge and stigma about suicide when providing care to suicidal patients.

**Methods:**

An anonymous web-based survey was implemented through the software REDCap. Data were collected by snowball sampling. Participation was voluntary and participants had the ability to opt out at any time. The study was approved by the University Ethics Committee. The survey consisted of the Informed Consent Form (ICF), Sociodemographic Questionnaire, Scale of Myths, Beliefs and Attitudes About Suicide (SMBAS) - which evaluates stigma about suicide trough true or false questions. We also included the Rating Scale for Countertransference (RSCT) which evaluates the main emotional responses towards suicidal patients, divided in approach, indifference or rejection. Other questionnaires were included for future research, beyond the scope of this study.

**Results:**

From 210 respondents, 179 (85.2%) completed the questionnaire. Sociodemographics: 108 (60.3%) were female; 166 (92.7%) were self-declared white-colored skin; The mean age was 37.22 (*SD* = 12.33), with 6 (0 to 48) median years of professional life [65(36.3%) were medical residents; 112(62.6%) were already specialists, 54(48.2%) of those declared to be psychiatrists]. Psychiatrists had highest rate of correct answers (*M = 28,96*, *SD* = 1,84) in SMBAS when compared with non-psychiatrists (M = 27,86, SD = 2,39 , *p* = 0,008); Psychiatrists presented more emotional responses of interest (M = 2,58, SD 0,68, *p* < 0,001), solidarity (M = 2,81, SD 0,45, *p* < 0,001) and desire to help (M = 2,83, SD 0,38, *p* = 0,010). Non-psychiatrists presented more emotional responses of hostility (M = 0,06, SD 0,25, *p* = 0,040) and distance (M = 0,32, SD 0,56, *p* = 0,002). Psychiatrists presented more approach (p=0,03) and non-psychiatrist indifference (p=0,03).

**Conclusions:**

We find preliminary evidence that psychiatrist present higher knowledge around suicide theme, having lower stigma around suicide. Psychiatrists also present more countertransference of approach and less of indifference.

**Disclosure of Interest:**

None Declared

